# The link between reported cases of COVID-19 and the Infodemic Risk Index: A worldwide perspective

**DOI:** 10.3389/fsoc.2022.1093354

**Published:** 2023-01-17

**Authors:** Federico Pilati, Riccardo Gallotti, Pier Luigi Sacco

**Affiliations:** ^1^Università IULM, Milan, Italy; ^2^Bruno Kessler Foundation (FBK), Trento, Italy; ^3^University of Studies G. d'Annunzio Chieti and Pescara, Chieti, Italy

**Keywords:** COVID-19, Twitter, Infodemic Risk Index, selective exposure, content moderation

## Abstract

In this brief report we followed the evolution of the COVID-19 Infodemic Risk Index during 2020 and clarified its connection with the epidemic waves, focusing specifically on their co-evolution in Europe, South America, and South-eastern Asia. Using 640 million tweets collected by the Infodemic Observatory and the open access dataset published by Our World in Data regarding COVID-19 worldwide reported cases, we analyze the COVID-19 infodemic vs. pandemic co-evolution from January 2020 to December 2020. We find that a characteristic pattern emerges at the global scale: a decrease in misinformation on Twitter as the number of COVID-19 confirmed cases increases. Similar local variations highlight how this pattern could be influenced both by the strong content moderation policy enforced by Twitter after the first pandemic wave and by the phenomenon of selective exposure that drives users to pick the most visible and reliable news sources available.

## Introduction

The COVID-19 pandemic crisis has turned the issue of (mis)information creation and circulation into a major cause of public concern. As the crisis unfolded, it has become clear that the actual rise of COVID-19 infections has been anticipated by large waves of potentially unreliable information that sowed mistrust and confusion in the public opinion (Gallotti et al., 2020). As people's behavioral response to a pandemic crisis is a crucial factor of success (or failure) of public health prescriptions (such as wearing masks), circulating misleading information and undermining the credibility of public health authorities can cause considerable damage and disrupt to a large extent the effectiveness of policy measures.

Such a loop between information acquisition and processing and adoption of more or less effective health-related behaviors may be at the core of different country performances in mitigating the outcomes of the global pandemic. For example, the spread of click-bait content or the prevalence of non-specialist, misleading opinions over those of scientists and public institutions on key public health matters are two dangers that are looming behind the COVID-19 communicational crisis (Damian and Gallo, 2020; O'Connor and Murphy, 2020). A key challenge that the pandemic poses to our society is therefore ensuring that correct, easily digestible information is received by citizens as a reliable guide to health-preserving choices (Zarocostas, 2020). Social media platforms are fully part of this process. As it has been shown, online conversations have a big impact upon the construction and perception of social reality, leading people to experience emotions without awareness (Kramer et al., 2014) and influencing the opinions of millions (Bond, 2012).

A fundamental key aspect of infodemic waves—i.e., an overabundance of information that makes it difficult for the majority of the public opinion to distinguish between reliable and unreliable sources—is their enormous pervasiveness both in news media and in the public's search for information (Eysenbach, 2002, 2009). When the COVID-19 crisis struck, the whole media ecosystem underwent an initial shock. Being a very powerful public attention pointer, COVID-19 quickly became the central topic of global conversations at any scale (Sacco et al., 2021). However, the rewards associated to the promotion of viral content by media outlets (Bakir and McStay, 2018), and the consequent incentives related to the trading of highly visible digital content for advertising and persuasive communication (Graham, 2017), has inevitably pushed the creation and circulation of sensationalistic, unreliable information able to capture the attention of large numbers of online users (Donovan, 2020). As a result, in the first months of 2020 a huge number of scientific hoaxes flourished and were disseminated on the web (McGinty and Gyenes, 2020).

Nevertheless, after the initial shock, extended surveys have partially changed the whole picture, showing how, faced with a serious crisis and potentially deadly threats, people were compelled to search and trust the information held by news sources considered reliable and familiar (Nielsen et al., 2020; Altay et al., 2022). Our research seems to confirm these findings. By analyzing a very large and heterogeneous dataset our results reveal the existence of a general negative correlation between the Infodemic Risk Index and reported COVID-19 cases, when considered both at global and regional scale. Significant local variations are however observed across different geographical macro-areas affected by the pandemic. Indeed, comparing the cases of Europe, South America, and South-eastern Asia, we have been able to highlight different co-evolutionary paths according to different local conditions.

## Methods

### Data collection

This article is based on the analysis of Tweets collected across 187 countries between 22nd January and 31st of December 2020. We automatically collected Twitter data from the Twitter Streaming API by selecting tweets containing terms associated with the COVID-19 epidemic, the virus that causes it and the city where it was first discovered (coronavirus, ncov, #Wuhan, COVID19, COVID-19, SARS-CoV-2 and COVID). The Streaming API limits our analysis to a random sample of 1% of the total number of tweets circulating. This limit has been reached, for the keywords we selected, on February 25th 2020. Starting from that date, only a random subsample of about 4M tweets/day are collected. All tweets are then processed in order to identify the country of origin and the type of news circulating. The identification for the country of origin is straightforward for a small fraction of ~0.8% of the total, for which the user shares the coordinates of the location from which the tweet has been posted. To extend this identification, we consider the user's self-defined location and derive the user's area of origin through a geocoding service. After specific filtering, this method allows us to associate about 50% of all tweets to a country of origin. Our data collection is possibly affected by a selection bias, as Twitter's user population skews the analysis toward well-educated males. In addition, by cross-checking with the news reliability database, this bias can be further exacerbated by an overrepresentation of users tweeting in English, as English web domains are better classified in the databases we aggregated. However, in Gallotti et al. (2020) it has been shown how, regardless of these possible biases, our methodology is appropriate to study the evolution of the COVID-19 infodemic in both time and space. In particular: i) the recall rate of tweets associated with the COVID-19 topic is higher than 16% and probably ranging in the 40%-60% range in the earlier days of the pandemic; the temporal patterns observed on the tweets whose provenience is from the U.S.A. as identified *via* geocoding services match those observed from tweets whose exact coordinates fall within the American territory: the results of the misinformation analysis appear robust to Twitter's policies to promote authoritative content by prioritizing the visibility of official sources.

### News enrichment

To identify the type of news circulating, we cross-check the URL shared in tweets with a database that aggregates different sources and categorizes the reliability of news web domains. To create the database we collected a list of manually checked web domains from multiple publicly available databases, including scientific and journalistic ones. Specifically, we considered data shared by the sources listed in:

– Zimdar, M. My fake news list went viral but made up stories are only part of the problem. The Washington Post (18 November 2016).
– Silverman, C. Inside the partisan fight for your news feed. BuzzFeed News (8 August 2017).
– Fake News Watch (2015). Retrieve from: https://web.archive.org/web/20180213181029/http://www.fakenewswatch.com/
– Politifacts guide to fake news and what they peddle. Politifacts.com (20 April 2017).
– The black list. La lista nera del web. Bufale.net (2018). Retrieve from: https://www.bufale.net/the-black-list-la-lista-nera-del-web/
– Starbird, K., Arif, A., Wilson, T., Van Koevering, K., Yefimova, K., and Scarnecchia, D. (2018). Ecosystem or Echo-System? Exploring Content Sharing across Alternative Media Domains. *Proceedings of the International AAAI Conference on Web and Social Media*, 12(1). https://doi.org/10.1609/icwsm.v12i1.15009
– Nielsen, R. K., Fletcher, R., Cornia, A., and Graves, L. (2018). Measuring the reach of “fake news” and online disinformation in Europe. (https://reutersinstitute.politics.ox.ac.uk/our-research/measuring-reach-fake-news-and-online-disinformation-europe)
– Grinberg, N. et al. Fake news on Twitter during the 2016 US presidential election. Science 363, 374–378 (2019).
– Media Bias/Fact Check (https://mediabiasfactcheck.com/).

We found a total of 4,988 domains, reduced to 4,417 after removing hard duplicates across databases. Note that a domain is considered a hard duplicate if its name and its classification coincide across databases.

A second level of filtering was applied to domains which are classified differently across databases (e.g., xyz.com might be classified as FAKE/HOAX in a database and as SATIRE in another database). To deal with these cases, we adopted our own classification method, by assigning to each category a “Harm Score” between 1 and 9. When two or more domains were soft duplicates, we kept the classification with the highest Harm Score, as a conservative choice. This phase of processing reduced the overall database to 3,920 unique domains.

The Harm Score classifies sources in terms of their potential contribution to the manipulative and mis-informative character of an infodemic. As a general principle, the more systematic and intentionally harmful the knowledge manipulation and data fabrication, the higher the Harm Score (HS).

A third level of filtering concerned poorly defined domains, e.g., the ones explicitly missing top-level domain names, such as.com.org etc, as well as the domains not classifiable by means of our proposed scheme. This action reduced the database to the final number of 3,892 entries.

### Infodemic Risk Index

The Infodemic Risk Index (IRI) represents an estimate of the relative exposure of users to unreliable information. To estimate the exposure to unreliable news (Eu), we aggregate the number of followers who are potentially reached by tweets containing unreliable news. Conversely, the exposure to reliable news (Er), is obtained by aggregating the number of followers potentially reached by tweets containing reliable news. The IRI is finally computed as IRI = Eu/(Er+Eu). See Gallotti et al. (2020) for further details, in particular on how analyzing an indirect measure of exposure using followers is largely equivalent to estimating exposure using actions (retweets, replies, quotes).

### Correlation and regression analysis

The regressions presented in this article have been made using the *statsmodels* python library. We performed logarithmic regressions [y = log(x)], as this curve has been seen to maximize the Akaike Information Criterion against possible alternative forms (linear, power law, logistic). Akaike is normally chosen since the alternative curves are characterized by different numbers of parameters. Curves with more parameters naturally adapt better to data, but are more prone to overfitting. Akaike considers this in a weight that accounts for the likelihood of a model considering the degrees of freedom. For this data, the Akaike weights were: logarithmic 4.933530e-01; linear 2.959338e-01; logistic 1.053566e-01; power law 5.690409e-136. The Spearman correlations and associated *p*-values are computed using the *scipy* python library. The Spearman correlation has been chosen as it is robust in the analysis of quantities ranging across several orders of magnitudes.

## Results

Finding 1: World countries most affected by COVID-19 have lower infodemic risk.^1^

**Figure 1:**
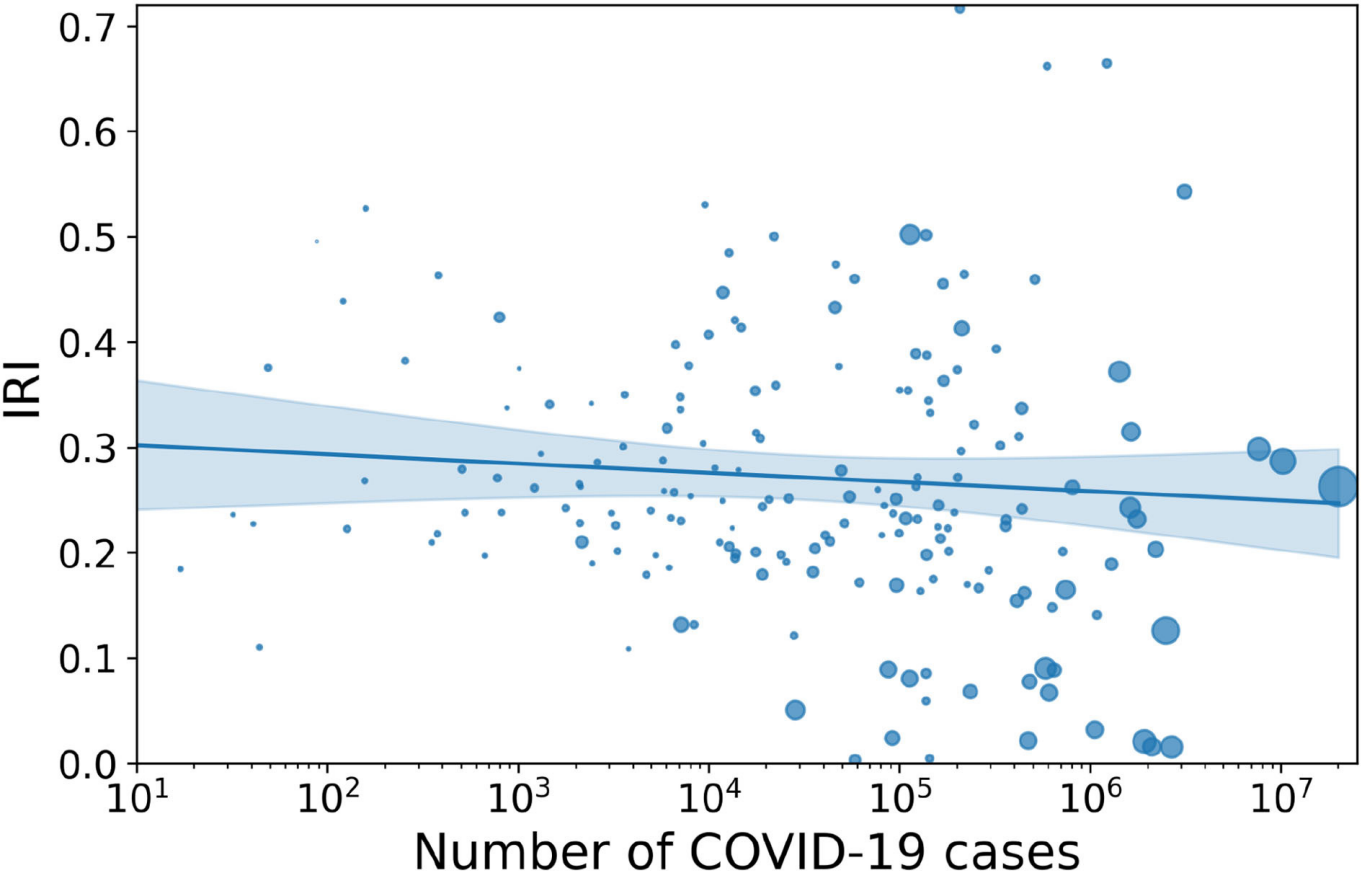
Correlation between IRI and total number of COVID-19 cases worldwide. Description: scatterplot showing a moderate but significant anti-correlation between COVID-19 cases and IRI across the 177 countries considered (Spearman-r is −0.16 and the p-value is 0.033). The correlation is strongly reduced as compared to the early days of the pandemic as analyzed in Gallotti et al. (2020), where Spearman-r was −0.33 and the *p*-value is 0.0009. The regression curve shown here and in all graphs is a logarithmic regression, which was selected using an Akaike criterion against a linear and a logistic regression. The shaded area represents the 95% confidence interval, and the size of the circle represents the number of Tweets collected. Two countries (Peru and Kosovo) are not visualized in the plot as they have IRI >0.9.

At a worldwide level (see Figure 1), we found a weak anticorrelation between reported cases of COVID-19 and index of infodemic risk. This link is all the more evident as the size of the countries considered in the sample increases: in fact, a larger size of the population implies a larger number of cases and also a larger statistical base to accurately and extensively count the impact of COVID-19.

Finding 2: The negative correlation between IRI and cases in South-Eastern Asia appears to be strong. The Spearman-r index is equal to −0.42 and the associated p-value is 0.23.

**Figure 2:**
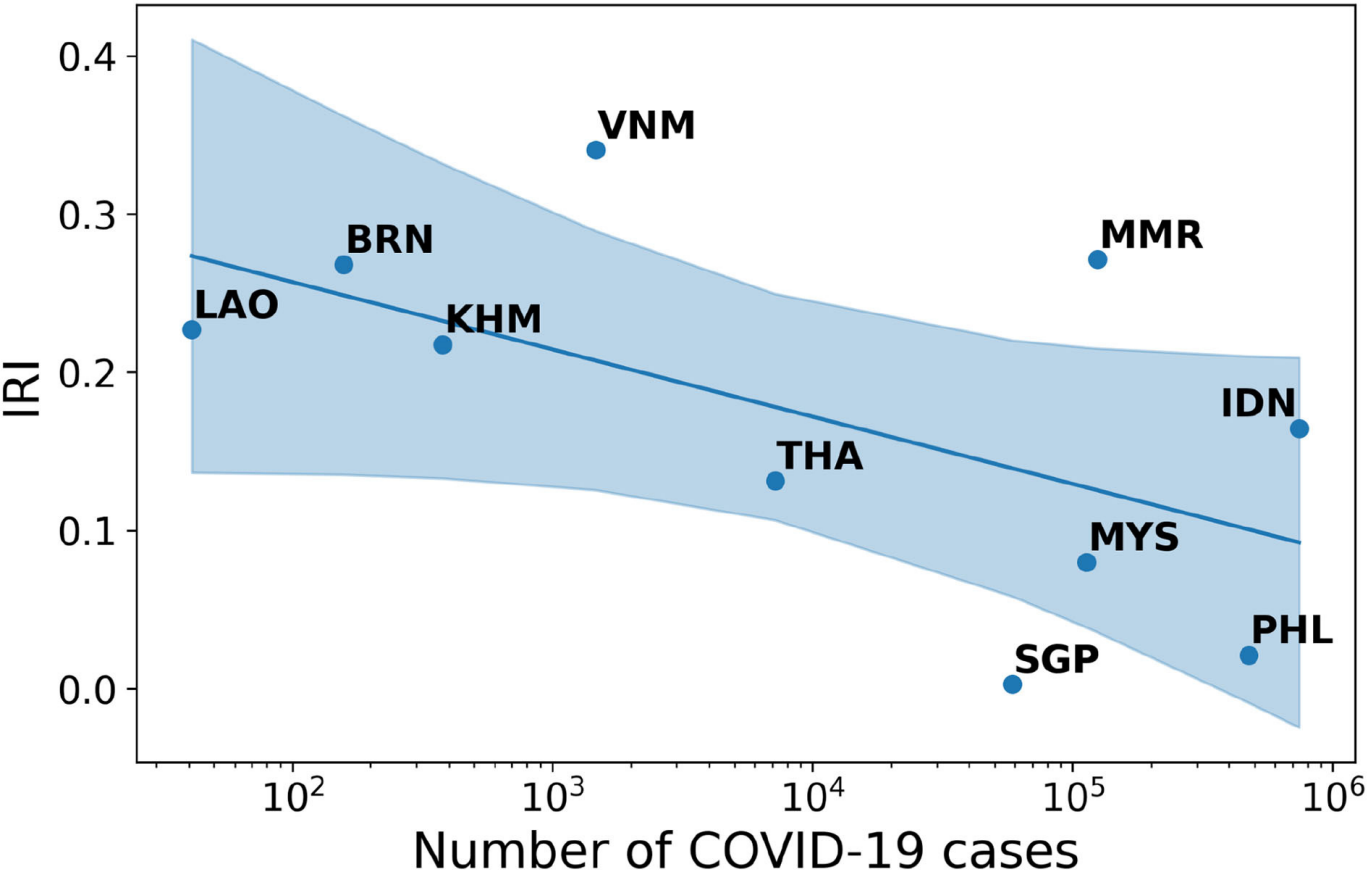
Correlation between IRI and number of COVID-19 cases in South-Eastern Asia.

South-Eastern Asian countries present from the beginning of the pandemic a marked difference in the pattern of infodemic risk. The IRI directly reflects the extent to which each single state in the region has been exposed to the pandemic waves (see Figure 2). Once the initial crisis stabilizes and the cases increase all over the region, even in the less risky countries there is a general increase of possibly harmful contents circulating in the Twittersphere (see Supplementary material).

Finding 3: The negative correlation between IRI and cases in Europe is as strong as in South-Eastern Asia. The Spearman-r index is equal to −0.50 and the associated *p*-value is 0.007.

**Figure 3:**
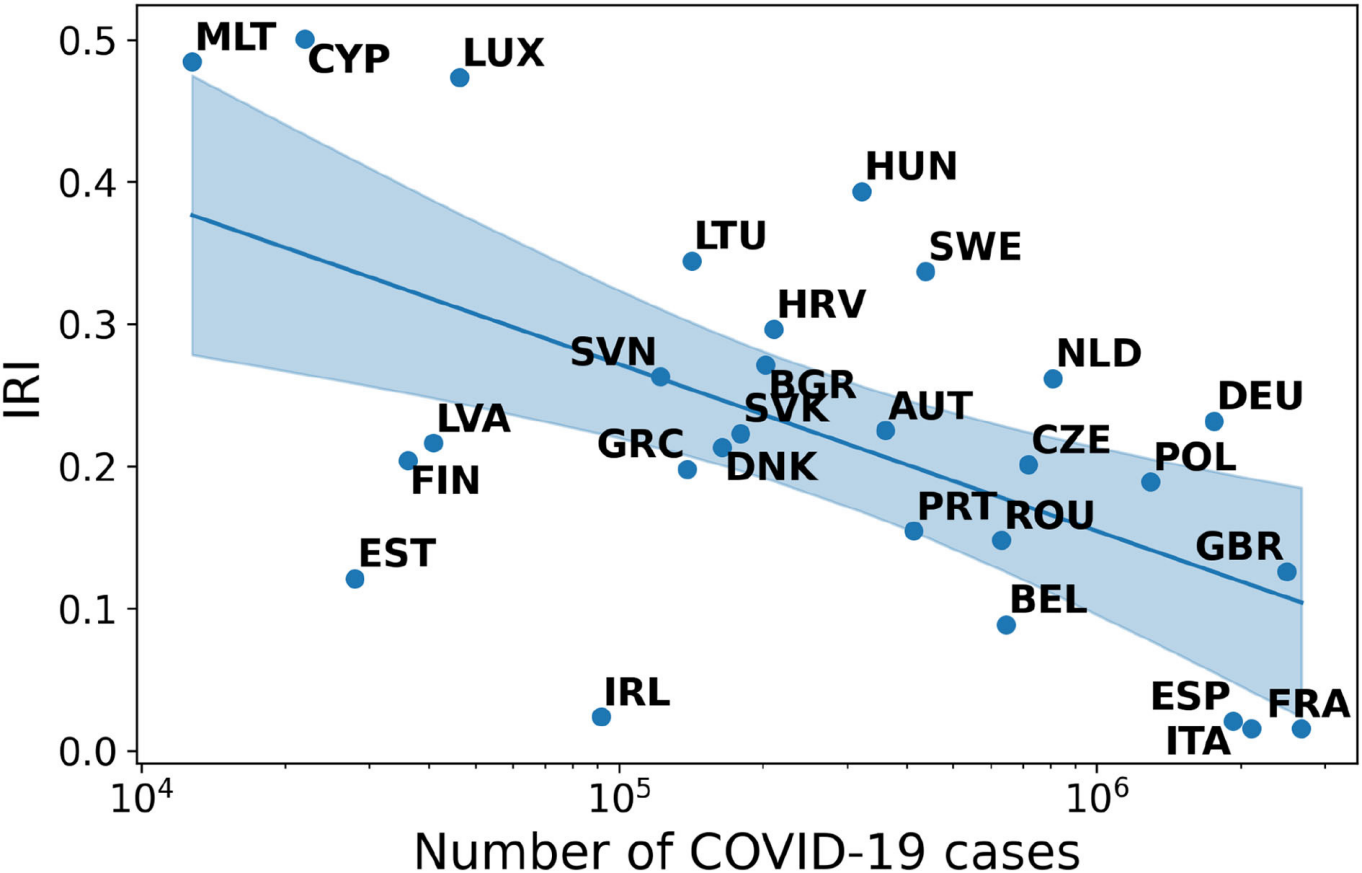
Correlation between IRI and number of COVID-19 cases in Europe.

Unlike Asean countries, in Europe the first wave of pandemic has been anticipated by a big, generalized surge of infodemic risk. As the number of cases increases, particularly in the most affected countries, the IRI visibly decreases (see Figure 3). This phenomenon could be observed in all countries that are gradually affected by the waves of infection and remains stable over time (see Supplementary material).

Finding 4: The correlation between IRI and cases in South America follows a positive pattern. The Spearman-r index is equal to +0.46 with an associated *p-*value of 0.13.

**Figure 4:**
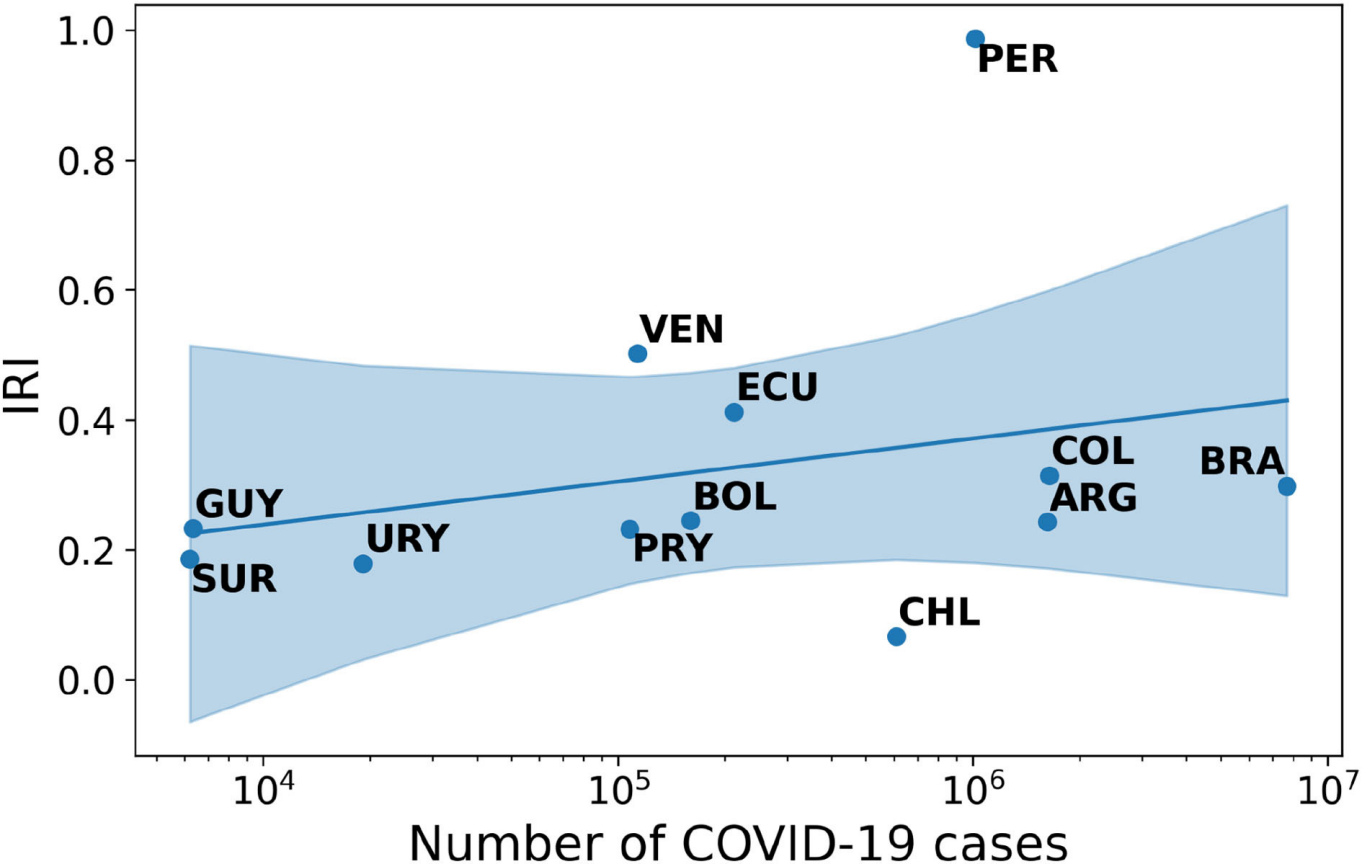
Correlation between IRI and number of COVID-19 cases in South America.

Finally, in South America the evolution of the relationship between IRI and COVID-19 cases directly reflects the trend of the pandemic (see Supplementary material). With an increase in the number of cases, countries experience an increase in disinformation content circulating on Twitter (see Figure 4).

## Discussion

From its very beginning, the COVID-19 crisis immediately raised concerns for risks generated by a possible infodemic of inaccurate information and how social media users would have dealt with it. In this brief report using the Tweets collected through the Infodemic Observatory's and the COVID-19 cases reported by Our World in Data, we analyzed the co-evolution of the Infodemic Risk Index and the pandemic from January 2020 to December 2020. Our findings describe a worldwide pattern of ongoing anti-correlation between Infodemic Risk Index and the reported cases of COVID-19. This relationship clearly emerges as the size of the countries in the sample increases: larger country population implies a larger number of cases and a larger statistical basis to assess the impact of IRI and COVID-19.

Each of the macro-areas examined shows a distinctive pattern of its own. South-eastern Asian countries present, from the beginning of the pandemic, a marked difference in infodemic risks as compared to other countries. Furthermore, their IRI directly reflects a specific country's exposure to the pandemic waves. However, once the initial crisis stabilizes and the cases increase all over one region, even in the less risky countries there is a general increase of possibly harmful contents circulating in the Twittersphere. For example, unlike the Asean countries trends, in Europe the first wave of pandemic is preceded by a big, generalized surge of infodemic risk. As the number of cases increases, however, and particularly in the most affected countries, the IRI visibly decreases. This phenomenon could be observed in all countries that are gradually affected by the waves of infection and remain stable over time. Finally, in South America the relation between IRI and COVID cases is proportional to the trend of the pandemic: as countries experience an increase in the number of cases, a corresponding increase in misleading content circulating on Twitter follows.

The negative correlation trend identified at a global scale can be partially explained by two different characteristics of the online and communication environments recorded since the onset of the pandemic. The first is the so-called selective trust or selective exposure phenomenon observed by Altay et al. (2022). Our data support such a theory: as the pandemic grows, citizens and the broader media ecosystem seem to be driven to pay more attention to reliable sources such as mainstream media. This effect could be further strengthened by an information cascade phenomenon, which is very common in Twitter. Indeed, the knowledge communities related to COVID-19 that populate Twitter are extremely hierarchical and mutually disconnected (Sacco et al., 2021). Therefore, a shift toward more reliable sources by the top and middle influencers of these communities could trigger a cascade effect on the entire flow of information regarding COVID-19.

Another possible reason behind the IRI decrease is a stronger focus on moderating conversations about COVID-19 by social media. This process may have been accelerated by the U.S. election—which triggered several monitoring projects regarding the politicization of COVID-19 issues (Chen et al., 2021)—with possible spillovers worldwide. For example, in the case of Indonesia, we could observe how most of the trolls and bots polluting the conversation in the early months of the pandemic were promptly removed from Twitter (Sacco et al., 2021).

As for the differences in patterns across the macro-regions, they may depend on two different factors. The first, and most obvious, are cultural differences that determine media consumption habits and attitudes toward the pandemic. In fact, as suggested by Gelfand et al. (2021), different cultures at national levels may have a big influence upon individual behavioral responses to COVID-19. A second factor relates to the politicization of the COVID-19 topic, and this would explain, for example, the positive correlation trend in South America. Indeed, a direct consequence of information manipulation strategies used by populist leaders, such as Bolsonaro (Ricard and Medeiros, 2020), can reinforce the sharing of misinformation. On the contrary, the bottom-up movements of fact-checking emerged in countries such as South Korea (Chang et al., 2021) could have impacted in its circulation reduction.

To sum up, our findings advance scientific literature on the infodemic in two different and complementary ways. First, as claimed by other empirical measures of Twitter (Yang et al., 2021), our results highlight how the effort on content moderation might have helped to drastically reduce the spread of COVID-19 misinformation. Second, our report supports the idea that when social media users face high health risks these very same users are compelled to search and trust information held by reliable news sources (Nielsen et al., 2020; Altay et al., 2022).

Finally it is important to conclude with a postilla regarding the methodological limitations of our research. As it is well known, the demographics of Twitter users are biased toward well-educated males (65 percent of Twitter users) between the ages of 18 and 34 (58 per cent of Twitter users, according to Statista GmbH). Our results therefore have to be interpreted keeping such demographic limitations in mind. Another important limitation is the necessarily restricted choice of hashtags which, although carefully designed, inevitably miss those parts of the social media flow that are not tagged according to the most common signifiers. However, it is important to consider that to the current state of knowledge there is no way to build a potentially unbiased, representative sample of the public opinion at the regional, national or global level, and to track its time evolution for relatively long periods. It will however be important to expand these methods to cover several social media at once, whose combined demographics and trend topics allow the coverage of different portions of the public opinion.

Our results are just a first step in understanding the loop between communications and the COVID-19 epidemic. Nevertheless, the recorded decrease for the Infodemic Risk Index as COVID-19 cases rise implies that pandemic-related misinformation can be successfully dealt with and that understanding the social and behavioral mechanisms behind its diffusion are essential building blocks for a successful misinformation curbing strategy.

## Data availability statement

Publicly available datasets were analyzed in this study. This data can be found here: https://covid19obs.fbk.eu/#/.

## Author contributions

FP, RG, and PS designed the research and interpreted the results and wrote the brief report. RG collected and analyzed the data. All authors contributed to the article and approved the submitted version.

## Footnotes

^1^To test the increased correlation of IRI vs Number of Uncorrelated Cases without rescaling by local population we repeatedly (2000 times) sample 100 random countries among those analyzed that had population data correctly indicated by the OECD for the year 2020. In figure “IRI vs. *p-*value” of the Supplementary material we plot the p-value of the Spearman correlation for P.C. and total cases against the Infodemic Risk Index. In only 12.5% of the samples the p-value is smaller using the per-capita number of cases, while in 24.2% of samples the p-value associated with the total number of cases is lower than 0.05.
