# Supplementary figures and images for: The link between reported cases of COVID-19 and the Infodemic Risk Index: A worldwide perspective
Source: Front Sociol. 2023 Jan 17;7:1093354. doi: 10.3389/fsoc.2022.1093354 (PMC9888028; doi:10.3389/fsoc.2022.1093354)

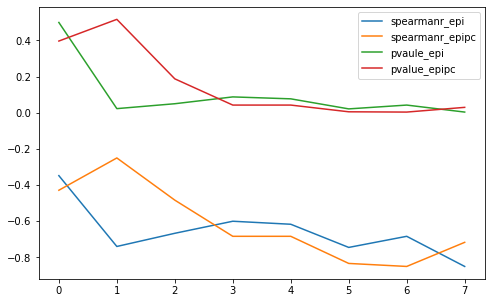

Supplement: Supplementary file 1 [file Image_1.PNG]

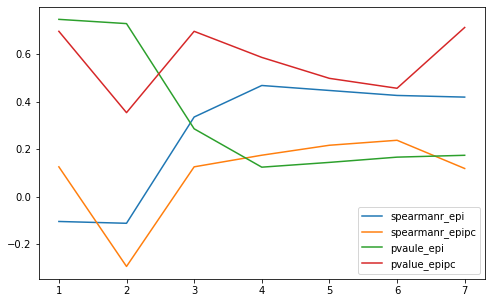

Supplement: Supplementary file 2 [file Image_2.PNG]

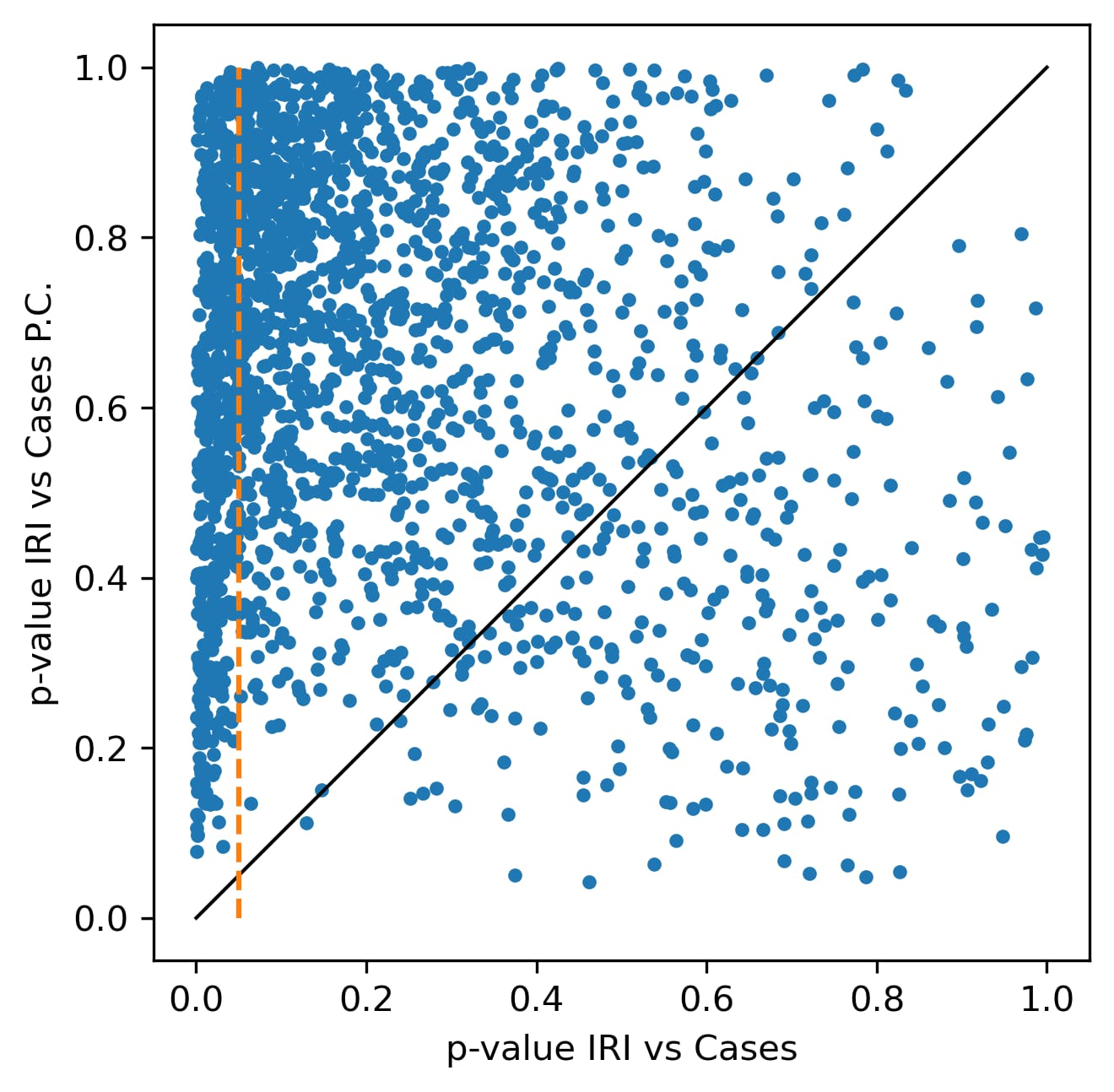

Supplement: Supplementary file 3 [file Image_3.JPEG]

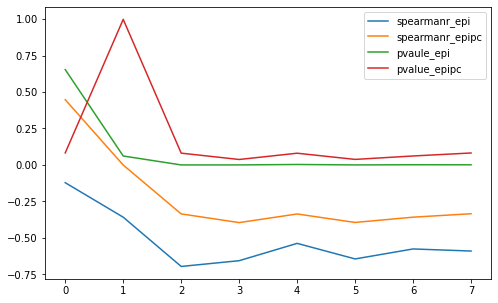

Supplement: Supplementary file 4 [file Image_4.PNG]
